# The Challenges to Improve Farm Animal Welfare in the United Kingdom by Reducing Disease Incidence with Greater Veterinary Involvement on Farm

**DOI:** 10.3390/ani3030629

**Published:** 2013-07-10

**Authors:** Philip R. Scott

**Affiliations:** Department of Veterinary Clinical Sciences, Roslin Institute and the University of Edinburgh, Easter Bush Veterinary Centre, Roslin, Midlothian, EH25 9RG, UK; E-Mail: Philip.r.scott@ed.ac.uk

**Keywords:** animal welfare, disease prevention, pain, analgesia, treatment, health plans

## Abstract

**Simple Summary:**

Sick cattle and sheep are often treated by farmers without prior veterinary examination and, as a consequence, incorrect diagnoses and inappropriate therapies are common, but these failings largely go undetected and unreported. Many farmers maintain that market forces render veterinary care of individual sick sheep and cattle too expensive. Delays in requesting veterinary attention are not uncommon causing unnecessary animal suffering and a poorer outcome. Incidence rates of endemic diseases in the United Kingdom are too high, causing animal welfare concerns, but these could be reduced by the implementation of proven veterinary flock/herd health programmes.

**Abstract:**

The Cattle Health and Welfare Group of Great Britain report (CHAWG; 2012) lists the most important cattle diseases and disorders but fails to fully acknowledge the importance of animal mental health and; in so doing; misses the opportunity to further promote animal welfare. There are effective prevention regimens; including vaccination; husbandry and management strategies for all ten listed animal health concerns in the CHAWG report; however control measures are infrequently implemented because of perceived costs and unwillingness of many farmers to commit adequate time and resources to basic farm management tasks such as biosecurity; and biocontainment. Reducing disease prevalence rates by active veterinary herd and flock health planning; and veterinary care of many individual animal problems presently “treated” by farmers; would greatly improve animal welfare. Published studies have highlighted that treatments for lame sheep are not implemented early enough with many farmers delaying treatment for weeks; and sometimes even months; which adversely affects prognosis. Disease and welfare concerns as a consequence of sheep ectoparasites could be greatly reduced if farmers applied proven control strategies detailed in either veterinary flock health plans or advice available from expert veterinary websites. Recent studies have concluded that there is also an urgent need for veterinarians to better manage pain in livestock. Where proven treatments are available; such as blockage of pain arising from ovine obstetrical problems by combined low extradural injection of lignocaine and xylazine; these are seldom requested by farmers because the technique is a veterinary procedure and incurs a professional fee which highlights many farmers’ focus on economics rather than individual animal welfare.

## 1. Introduction

The Animal Welfare Act 2006 (England and Wales) includes a duty of care to provide for the needs of protected animals for which humans have permanent or temporary responsibility [1]. Article 9(2)(e) of the Animal Welfare Act 2006 sets out an animal’s “need to be protected from pain, suffering, injury and disease.” 

### 1.1. Farm Animal Welfare Council’s Five Freedoms

The author is recognised as a veterinary specialist by the European College of Small Ruminant Health Management and the European College of Bovine Health Management and has 35 years’ experience of farm animal practice in the UK. The diseases and conditions discussed in this article have been selected because they commonly occur and cause obvious welfare concerns as defined by the Farm Animal Welfare Council’s Five Freedoms [2] (Table 1) but are seldom presented to veterinary practitioners for diagnosis and treatment. Without a specific diagnosis, well-proven prevention and control strategies are not adopted and the resultant high disease prevalence rates adversely affect animal welfare. The FAWC “Five Freedoms” provide a comprehensive template that incorporates the different elements that define welfare state [3].

**Table 1:** The Farm Animal Welfare Council’s Five Freedoms (FAWC, 1993 [2]).

**Freedom from hunger and thirst**	by ready access to fresh water and a diet to maintain full health and vigour
**Freedom from discomfort**	by providing an appropriate environment including shelter and a comfortable resting area
**Freedom from pain, injury or disease**	by prevention or by rapid diagnosis and treatment
**Freedom to express normal behaviour**	by providing sufficient space, proper facilities and company of the animals’ own kind.
**Freedom from fear and distress**	by ensuring conditions and treatment which avoid mental suffering

Early recognition of a disease challenge and correct diagnosis that lead to rapid and effective treatment are key to keeping farm animals healthy and thus protecting their welfare [4].

### 1.2. Differentiating Animal Health from Animal Welfare

In FAWC’s opinion, animal welfare encompasses both physical and mental health [4]. Good physical health is essential for good welfare, but is not sufficient because it does not necessarily lead to good mental well-being [4]. Conversely, poor productivity, e.g., infertility [5], may be indicative of an underlying disease or management problem but may not always cause suffering. When assessing any potential animal welfare concern, it is necessary to consider the extent of poor welfare, the intensity and duration of suffering, the number of animals involved, the alternatives available, and the opportunities to promote well-being [4]. An animal that suffers from poor welfare is not the same as an animal that suffers pain whereas an animal that suffers pain necessarily means that its welfare is compromised [6].

Veterinary surgeons are experts in animal health but it is the consideration of the mental well-being of farm animals that differentiates animal welfare from animal health [4]. For example: when cereal-fed (“barley beef”) cattle are confined on slatted concrete floors at the highest permissible stocking density they may appear healthy and grow well but may not experience a good life, and some would reason that they may not have a life worth living because they have little space to exercise, are fed a monogastric animal’s ration likely to cause defective hoof horn growth and lameness, often there is no dietary roughage, and consequently they rarely ruminate. Use of these descriptive terms developed by the FAWC to describe the quality of an animal’s life, may be naïve and rather simplistic but have proven very helpful and direct when discussing disease prevention, management and treatment options with farmers. Further debate on this concept concludes “by endorsing an overarching principle that no animal should be unreasonably caused to be, or allowed to remain, in a position of having a prospective life worth avoiding” [7] but this is a much more complex viewpoint to present to farmers than the basic FAWC descriptors.

Of the Five Freedoms, freedom from pain, injury or disease by prevention or by rapid diagnosis and treatment most directly concerns the veterinary surgeon in farm animal practice. This paper will discuss the third of FAWC’s Five Freedoms (Table 1) and detail the identification and potential alleviation of pain in cattle and sheep, the common diseases causing pain, and examples of how the incidence of some common diseases can be reduced thereby improving farm profitability, and more importantly animal health and welfare.

## 2. Pain and Alleviation of Pain

### 2.1. Recognition of Pain

Pain has been broadly classified as either adaptive or maladaptive [8]. Adaptive pain increases the potential for survival by protecting the animal from injury and by promoting healing and is expected after surgical procedures on healthy tissue. By contrast, maladaptive pain is a disease created by pathologic processes that result in the persistence of pain long after the initiating cause(s) have been removed such as digit amputation for septic pedal arthritis [9].

Freedom from pain is often considered a major indicator of good animal welfare by the veterinary profession; mental health is a lesser consideration because it is much more difficult to quantify. Pain is typically assessed by changes in behaviour and demeanour, stance, lameness, lowered food intake, reduced use of the affected part, and behavioural signs (listed in Table 2). Ruminants experiencing pain often become subdued, spend more time lying down, spend less time eating and ruminating, and fail to clean the nostrils as frequently [9]. Interpretation of multiple behavioral responses as an aggregate indicator of animal wellness status instead of as individual outcomes is regarded as a more accurate measure of true state of animal pain or wellness status [10]. 

The occurrence of pain can generally be more reliably identified than its intensity. 

**Table 2:** Identification of pain in cattle and sheep.

Dullness, depression, lethargy Grunting, teeth grinding Inappetence, decreased rumination rate Increased respiratory rate Increased/reduced vocalisation Increased sensitivity (hyperalgesia) Attention/licking at site of pain.

Farm animal clinicians maintain that they can identify pain in sheep and cattle but there is great variation in pain scores attributed to many infectious diseases and traumatic injuries regularly attended by veterinary practitioners [11,12]. Furthermore, analysis of replies from a questionnaire survey concluded that there was an urgent need for veterinarians to better manage pain in livestock [13]. From a veterinary standpoint, inflammation can be more readily identified than pain, and is characterised by localized heat and swelling, increased sensitivity to palpation, and loss of function (e.g., lameness when part of the musculoskeletal system is inflamed). It is important to recognize inflammatory conditions because they are a major contributor to adverse animal welfare [14].

### 2.2. Alleviation of pain

Whilst steroids and NSAIDs may have benefits such as mitigation of pain, lessening of swelling, diminishing inflammation at the incision site and/or damaged tissues, and more rapid patient recovery after the procedure [9] there are no drugs currently approved for pain management in food animal species in the USA however several products are used in an extra-label manner [15]. Non-steroidal anti-inflammatory drugs (NSAIDs) inhibit the cyclo-oxygenase enzymes, and decrease peripheral and central prostaglandin production. In addition to reducing the inflammation that accompanies tissue injury, decreasing prostaglandin production attenuates the response of the peripheral and central components of the nervous system to noxious stimuli. Such a reduction in the response to pain can reduce the peripheral and central sensitisation induced by noxious stimuli, and reduce the pain experienced in response to subsequent noxious stimuli [16] In the UK NSAIDs, in conjunction with antibiotics, are licensed to treat respiratory diseases, mastitis and diarrhoea in cattle where they reduce the severity of clinical signs of disease but there is no specific licensed claim for analgesia in their data sheets. Flunixin is the only NSAID labeled for use in beef and dairy cattle in the USA and is indicated for the control of pyrexia associated with bovine respiratory disease and mastitis as well as for the control of inflammation associated with endotoxaemia [15]. Extrapolating the potential benefits of NSAIDs in these infectious diseases, veterinary surgeons and farmers frequently administer NSAIDs to cattle and sheep with other infectious diseases and trauma arising from dystocia however there is limited published evidence regarding their usefulness as analgesics in these situations [17]. Furthermore, there is little evidence for the use of NSAIDs in the treatment of trauma in recumbent cows, even though an expert panel concluded that NSAIDs were a key aspect of veterinary treatment of downer cows [17]. The widely held view by veterinary surgeons that NSAIDs are effective analgesics in farm animals is not always supported by published clinical evidence.

In discussions with clients many veterinary practitioners incorrectly describe NSAIDs as “pain killers” despite the lack of supporting scientific evidence for many diseases. As a consequence, many farmers mistakenly believe that NSAIDs significantly reduce pain and their administration overcomes most animal welfare concerns arising from situations where there has been either delayed detection and treatment of clinical disease or trauma such as excessive traction applied during assisted delivery of a calf or lamb. Consequently, many potential animal welfare concerns are simply treated with NSAIDs without adequate nursing provision such as adequate bedding materials, frequent rolling to prevent pressure sores in recumbent animals, and ready access to food and clean water.

Non-steroidal anti-inflammatory drugs have powerful anti-endotoxic properties [18] such that improved demeanour (Table 2) after treatment may not necessarily have resulted from reduction in pain and improved animal welfare only treatment of the primary infectious disease. Clinical improvement after NSAID administration in cattle with coliform mastitis [18,19,20,21] results because NSAIDs have specific actions against the endotoxin causing clinical disease [22] rather than reduction of pain *per se*.

Non-steroidal anti-inflammatory drugs have also been recommended to treat cows that have suffered dystocia where the annual incidence is reported to be as high as 9% in dairy herds [23]. However, some studies reported adverse effects, such as pyrexia and increased risk of metritis after NSAID administration [17], and NSAID treatment had no benefit on the occurrence of peripartal diseases or fertility parameters [24]. 

Non-steroidal anti-inflammatory drugs have been shown to reduce pyrexia for 6–24 hours [25,26,27,28], improve clinical signs [26,28], reduce lung pathology [25,28], and increase average daily weight gains [29] in calves with respiratory disease compared to untreated calves or calves treated only with antimicrobial drugs. Other studies, however, have found no significant differences when antibiotic treatment was initiated during the early stages of disease defined by significant pyrexia [30,31]. The major difference between these studies was the duration of clinical signs before treatments were administered; the more advanced the clinical signs the greater the response to NSAID injection. Certain studies have questioned the cost-efficiency of additional anti-inflammatory therapy in bovine respiratory disease [32]. The simple message to farmers must therefore be close supervision of susceptible cattle during recognized disease risk periods and early veterinary diagnosis and treatment. However, some pharmaceutical companies still actively promote whole group antibiotic therapy to treat bovine respiratory disease despite high treatment failure rates [33] and concerns over the development of antimicrobial resistance. 

The available perioperative trials of pre-emptive NSAID use in humans have yielded modest or equivocal results, and these may be due, in part, to controversy associated with the definition of pre-emptive analgesia and how to conduct the corresponding clinical trials [16]. Relative to control cows receiving no NSAID injection, ketoprofen did not alter the physiological measures, serum 3-OH butyrate concentrations (an indicator of energy balance), or measured behavioural outcomes in cattle undergoing surgery to correct left abomasal displacement [34].

Recent studies using the NSAID meloxicam have been shown it to be effective in mitigating some of the pain caused by castration and dehorning in calves [35] although not all reported studies show a significant benefit using pre-emptive NSAIDs [36]. A multimodal approach using local anesthetics, NSAIDs and, when possible, sedative analgesics, is recommended for the most effective reduction of pain response in cattle following dehorning [37].

## 3. Physical Injury

There are no published data on the value of treating calves with NSAIDs after dystocia despite significant tissue damage during delivery [17]. The NSAID meloxicam administered to cattle dairy cattle 24 hours after assisted parturition had no affect on appetite (dry matter intake), milk production or health events [38]. Animal welfare in this situation is better served by early veterinary attendance and correct treatment, possibly surgical delivery of the calf, rather than excessive traction and trauma followed by NSAID injection. 

Studies have shown a reduced pain response in calves disbudded by heat cauterization after NSAID administration in addition to effective local anaesthesia [39,40].

Flunixin meglumine, a commonly used NSAID in cattle, had no effect on the thresholds to noxious mechanical stimulation in lame sheep [41]. No analgesic response was detected after flunixin meglumine injection using algesimetry in sheep based on a leg withdrawal response to an electrical stimulus [42]. In a clinical situation, there was no significant effect of NSAID treatment on the time to recovery from lameness caused by footrot in sheep [43].

## 4. Disease Prevalence and Incidence Rates

FAWC considers freedom from pain, injury or disease to be of the utmost importance but the prevalence of many endemic diseases in farm animals in the UK is too high [4]. The lack of progress in dealing with animal welfare concerns is most evident in dairy cow lameness. Lameness prevalence figures in dairy herds have not improved over the past two decades with prevalence rates around 20 per cent [44] despite the fact that experts rate lameness and discomfort as highly important indices of poor welfare in dairy cows [45]. Studies have reported lameness prevalence as high as 49 per cent [46]. More than 75 per cent of animal welfare experts considered that at least 42 of 53 dairy farms in one study [47] needed to take action to reduce the prevalence of lameness, overgrown claws, swollen and ulcerated hocks, and injuries from the environment. The incidence of lameness in dairy herds in the UK remains unacceptably high because of basic management deficiencies such as poor cubicle design and inadequate foot-bathing [48] rather than the absence of specific knowledge or lack of appropriate therapies. Veterinary practitioners have an important role in educating clients how to recognize and respond to livestock pain [49].

The CHAWG of Great Britain published its first report of disease incidence data (Table 3) to help primary producers and the industry, including veterinarians and Government, set a framework so that progress can be gauged and reported on an ongoing basis [5]. While these data are far from complete, the report contains important information upon which veterinarians can base farm health management decisions, and thereby have a positive impact on animal welfare. 

**Table 3:** Ten most important health and welfare issues of beef and dairy cattle in Great Britain as reported by CHAWG (not ranked in order of importance).

Beef	Dairy
(In)Fertility	(In)Fertility
Mastitis	Mastitis
Infectious Bovine Rhinotracheitis	Lameness
Bovine Viral Diarrhoea	Bovine Viral Diarrhoea
Johne’s Disease (Paratuberculosis)	Johne’s Disease (Paratuberculosis)
Liver Fluke	Bovine Tuberculosis
Nutrition	Nutrition
Calf Pneumonia	Calf Pneumonia and scour
Calf Scour	Parasitic Gastroenteritis /Lungworm
Parasitic Gastroenteritis /Lungworm	Genetics

However, the CHAWG report is revealing in that the conditions listed in Table 3 represent the major production losses in the cattle industry. Several of the diseases listed, namely BVD, Johne’s disease (paratuberculosis), and liver fluke would not be considered major welfare concerns, and were not considered in a cattle pain survey of veterinary surgeons undertaken in 2006 [11]. BVD and paratuberculosis are estimated to cost the UK cattle industry up to £61 million and £13 million annually, respectively. Losses from fasciolosis (liver fluke) in 2011 were £23 million with up to 30% of cattle livers condemned at slaughter. Most cattle with fasciolosis still achieved target growth rates, showed no ill health but the infestation is considered important because of financial losses to the industry from condemned livers. Similarly, BVD infection causes no direct animal welfare concerns unless there is significant secondary bacterial infection causing overt secondary disease but is considered important because of production losses resulting from infertility, abortion and congenital abnormalities. Infection of growing cattle with BVD virus in many farms situations causes no clinical signs. Bovine tuberculosis (bTB) was estimated to cost the UK government over £80 million in 2011. Few, if any, culled cattle had bTB lesions causing serious illness; slaughter was undertaken for international trade reasons not cattle welfare. Whilst the economic consequences of disease are very important [5] animal welfare impact must not be ignored and disease must not be evaluated solely on a financial benefit:cost basis. 

## 5. Improving Animal Welfare by Prevention and Control of Specific Diseases—Examples

Cost-effective prevention strategies are listed (Table 4) for most of the conditions listed in CHAWG report (Table 3) and are briefly discussed for specific diseases. Diseases listed in this section are included as examples; detailing all control measures is out-with the scope of this article. 

**Table 4:** Some proposed key interventions for the ten most important diseases in beef cattle in Great Britain (not ranked in order of importance; CHAWG report [5]).

Diseases/problems	Key intervention
(In)fertility-dystocia	Genetics, management
Chronic mastitis	Management, dry cow therapy, culling
Infectious Bovine Rhinotracheitis	Biosecurity, vaccination, eradication
Bovine Viral Diarrhoea	Biosecurity, vaccination, test and cull policy, eradication
Johne’s Disease	Biosecurity, biocontainment, test and cull policy
Liver Fluke	Biosecurity, strategic treatments
Nutrition	Correct management
Calf Pneumonia	Housing, management, vaccination
Calf Scour	Management, dam vaccination
Parasitic Gastroenteritis /Lungworm	Grazing management, strategic treatments, vaccination (lungworm)

### 5.1. Bovine Neonatal Diarrhoea (Calf Scour)

Neonatal diarrhoea (scour) was the most common disease reported in young calves and the greatest single cause of death [5]. Despite the known causes in most cases, and proven efficacy of a vaccine administered before calving to control the important viral and bacterial enteropathogens, only 10–15% of UK beef cows are vaccinated to control calf scour; 85% to 90% of calves are thereby unprotected and susceptible to disease. Environmental hygiene, biosecurity and biocontainment, and many aspects of husbandry including cow nutrition during late gestation and passive antibody transfer are also important in the control and prevention of calf scour.

### 5.2. Bovine Respiratory Disease (Calf Pneumonia, Infectious Bovine Rhinotracheitis)

There are a large number of highly efficacious respiratory vaccines available but only 10–15% of at-risk growing cattle are vaccinated. Furthermore, recent surveys have estimated that approximately 60% of farm buildings used to house cattle over the winter months in the UK have inadequate ventilation. Once again, environmental hygiene, biosecurity and biocontainment, are also important in the control and prevention of respiratory disease.

### 5.3. Sheep Obstetrics

Pain management of small ruminants has historically been discounted [50]. However, significant improvements in sheep welfare have been demonstrated after sacrococcygeal extradural injection of 0.5–0.6 mg/kg lignocaine in ewes with obstetrical problems attended by veterinary practitioners [51,52]. These analgesic protocols have also demonstrated effective analgesia of extended duration [53,54,55,56,57,58,59] and represent a “leap forward in the alleviation of suffering in ewes” [60].

The scale of welfare concerns arising from obstetrical conditions is reflected in data collected over 11 years from 31 veterinary practices servicing clients with a total of 575,000 breeding sheep. Consistently few ovine obstetrical problems were attended annually over the study period (1996–2006) with veterinary treatment of only one of 2,300 sheep, and one caesarean operation per 5,700 ewes [61] when obstetrical problems of vaginal prolapse and dystocia that would benefit from expert assistance occur at a rate around 0.5 to 1 per cent, respectively (veterinary attention needed for every 50 to 100 breeding sheep). Hindson and Winter (2002) reported that many ewe deaths were undoubtedly related to mismanagement of obstetrical problems undertaken by farmers unwilling to pay for veterinary services [62]. Lambing difficulties dealt with by farmers account for up to 70 per cent of ewe deaths [63].

A survey of 95 farms with more than 79,000 breeding ewes reported that only 22 of 359 (6.1%) dystocia cases that could not be corrected by the farmer received veterinary attention; 65 sheep were humanely destroyed, while 272 ewes subsequently died presumably due to death of the lambs that could not be delivered with development of an overwhelming toxaemia [64]. The number of ewes with dystocia in a survey of 89,000 ewes was 4,313 (4.8%) with a mortality rate of 79.3% for farmer-assisted dystocia cases; only 289 ewes (6.7% of all dystocias) were presented to a veterinary surgeon [65]. While it could be reasoned that death removes any welfare concern in ovine dystocia cases, surgery undertaken by a veterinary surgeon achieves >98% success rate with normal maternal behaviour and appetite observed almost immediately following surgery [66]. The flock health plan for a 1,000 ewe flock (Table 5; adapted from [67]) applies well-researched methods for elimination and/or control of common sheep diseases and disorders all funded from within current farm costings for veterinary fees and medicines. 

### 5.4. Sheep Lameness

In 1994, the farmer-estimated prevalence of lameness in English flocks was 8.4% [68] and was similar a decade later at 10% [69]. Lameness prevalence was quoted between 9–15% in 2004 [70]. There are proven treatment, prevention and control measures for most of the important causes of lameness [70,71] however sheep are not treated early enough and suffer as a consequence of such delays [72]. For example approximately 15% of farmers do not catch and treat individual lame sheep [73]. In a study of septic arthritis in adult sheep, only five of 39 animals were presented for veterinary investigation within one week of the onset of non weight-bearing lameness; many of the other 34 sheep had extensive bony changes involving the infected joints indicating more than two months’ severe lameness before veterinary examination [74]. Similar duration of painful lesions was observed in a report detailing septic pedal arthritis [75], and elbow arthritis [76].

### 5.5. Sheep Scab

Sheep scab is a serious animal welfare problem caused by the skin parasite *Psoroptes ovis* which is now endemic throughout the UK [77,78] since de-regulation of compulsory dipping in 1992. A prevalence rate of 17% was recorded in Wales for 2003/04 [79], while a later study found a prevalence rate of approximately 24% [80]. In a study of Great Britain, the overall prevalence rate was reported to be 35% over a three years’ period between 2002/05 [81]. Despite the adverse consequences of sheep scab infestation during mid gestation on ewe body condition loss and lamb birthweight reported almost 20 years ago [82] many farmers do not take this problem seriously and 8.5% of farmers did not treat infected animals [83]. 

#### Sheep Flock Health Planning—An Example

A veterinary flock health plan to improve the welfare status of the sheep flocks proposed the introduction of husbandry practices, anthelmintic treatments, and vaccinations that could be achieved within industry costs of £3.50 per ewe per annum for veterinary fees and medicines [67]; an updated version is reproduced in Table 5. 

**Table 5:** Annual cost of operating a flock health plan for a 1,000 ewe lowland flock in the UK producing 1,500 lambs sold for slaughter by 5 month-old (adapted from [67]).

Chlamydophila vaccination (25% flock)	£500
Toxoplasmosis vaccination (25% flock)	£500
Clostridial diseases vaccination (all adult sheep)	£150
Sheep scab control (all purchased sheep—25% flock)	£200
Parasitic gastroenteritis (diarrhoeic sheep)	£200
Quarterly veterinary visits (1.5 hours at £60 per hour)	£360
10 dystocia cases (at veterinary surgery)	£450
10 prolapse cases (at veterinary surgery)	£200
Examine 10 sheep (misc. problems at surgery)	£100
Drugs	£200
Treat/prevent PGE (1,500 lambs; two treatments)	£300
Cutaneous myiasis control (soiled lambs only; 10%)	£60
Footbath chemicals (1,500 lambs; two treatments)	£300
TOTAL	£3,420

The proposed flock health plan tackled obstetrical problems which the authors considered the most important animal welfare concern but also included those diseases considered of major economic importance by industry. The estimated annual costs of gastrointestinal parasites in Great Britain were £84 million, £24 million for footrot, £8 million for sheep scab, £20 million for chlamydial abortion, and £12 million for toxoplasmosis [84]. 

## 6. Good Stockmanship and Effective Prevention Regimens

### 6.1. Stockmanship

The health and welfare of dairy cows are dependent upon the stockmen who handle, observe, and monitor them [85]. However, there is currently no direct surveillance of stockmanship [4]. It is often assumed that farmers and stockmen are competent but poor provision of the most basic animal husbandry tasks such as isolation of sick calves, ingestion of colostrum within six hours of birth, and the provision of fresh, clean water has been reported [86]. Veterinary practitioners report the incidence of lameness in dairy herds remains unacceptably high because of basic management deficiencies [48]. 

The accuracy of farmer diagnoses and selection of correct treatment(s) can rarely be determined because relatively few sick animals are examined by veterinary surgeons. Dairy cow mortality is an increasingly severe problem for the dairy industry in the United States [87]. Where data are available mis-diagnoses are common e.g., hypocalcaemia is a common metabolic disease of sheep with pathognomonic signs but only 38.6 per cent of ewes treated by the farmer survived [65] whereas the response to veterinary treatment is 100 per cent [88,89]. 

### 6.2. Knowledge Application

Whilst practical nutrition guidelines have been developed for use by veterinary practitioners to correct the adverse effects of ewe underfeeding on lamb birthweight [90], and subsequent perinatal lamb mortality [91,92], there was no reduction in perinatal lamb mortality in the following 15 years [62]. Communication is clearly an important issue in disease control [93]. In a study asking why sheep farmers did not request veterinary assistance for dystocia cases; 33% of respondents quoted excessive professional fees, while 31% considered themselves as competent as their veterinary surgeon in such matters [65]. In this respect, competence of veterinary graduates in obstetrical procedures is essential but the ability to successfully perform a caesarean operation remains an essential skill at graduation.

Many husbandry practices and postoperative case management can be improved with attention to pain management [50] although there is little evidence that pain management is associated with increased production outcomes [94]. The recent FAWC report on education, communication and knowledge application [95] calls for Government and industry to overcome barriers to the transfer and implementation of knowledge by provision of authoritative and accessible advice and supporting commercial initiatives in this context to ensure recognition of abnormal conditions in animals, diagnosis of causes and correct treatment. Several websites have provided farmers free access to expert veterinary advice with particular emphasis on animal welfare concerns for many years [96]. Webinar delivery has proven a cost-effective and popular method of delivering continuing profession development to many professions, including veterinary surgeons, and this format could readily be developed to support farmers’ needs particularly in geographically remote areas. 

### 6.3. Farm Animal Health Planning

According to the Department of the Environment Food and Rural Affairs, around 60% of UK livestock farmers had a written farm animal health plan in 2012, but only 41% of farmers used their health plan on a routine basis to impose disease management decisions [5]. Howard (2004) also reported a lack of veterinary input into the health and welfare on dairy farms [86]. Fewer than 10% of agricultural holdings in Scotland accepted funding for veterinary involvement in farm health planning offered through the Animal Welfare Management Programme of the Scottish Government [97]. This figure is similar to results of a survey undertaken in 1999 which revealed that less than 10 per cent of Welsh farmers employed veterinary services in any consultative capacity [65]. 

Biosecurity measures can be undertaken at farm level to prevent introduction of common diseases and such measures could do much to improve the health, productivity and welfare of sheep yet many UK sheep farmers take no animal health precautions either when introducing purchased animals to their flocks or at farm boundaries [98]. 

The Sheep Veterinary Society website document on the welfare of sheep in the UK states that “all flocks under the care of a veterinary practice should receive at least one veterinary visit per year when routine management and treatment of the flock would be discussed [99]. In the event of a disease outbreak or incident not anticipated by the annual visit, then the flock may require a further visit for disease diagnosis and supply of medicines” [99]. It is this author’s experience that one veterinary visit during the annual production cycle will not address all health and welfare issues that arise in flocks that may total several thousands of sheep. This opinion is supported by the continuing problem of sheep scab where only 57% of diagnoses were made by a veterinary surgeon [100].

The CHAWG report [5] contains the disturbing statement that about 240,000 adult cattle in GB die each year of unknown or unrecorded causes on farms. It is unclear whether these cattle died or were euthanased for welfare reasons and more information is urgently needed to address this situation because death is likely to have followed several days’ severe illness; there are relatively few infectious diseases that kill cattle within 24 hours. Gregory (2011) reports recent studies which are claimed to highlight welfare issues pertaining to the prognosis for cows following replacement of a prolapsed uterus whereby “allowing a cow to take four or more days to die when the prognosis is poor is hard on the cow” [101]. A recent study found that more sheep died on farms than were culled for poor condition or suspected disease [102]. Animals must be humanely euthanased on farms and not simply left to die.

## 7. Conclusions

Reducing disease prevalence rates by active veterinary herd and flock health planning and increasing veterinary care of many problems presently “treated” by farmers would greatly improve individual animal and overall flock/herd health and welfare. Administration of a NSAID does not address all the welfare needs of sick and injured farm animals. There are well-researched flock health plans published in the literature to address the major sheep diseases and welfare concerns. There are also excellent on-line information packages which are freely available to veterinary surgeons and farmers. The CHAWG report [5] is not sufficiently ambitious in setting targets for improving animal welfare; financial considerations fail to address the important animal health concerns. While it is common for producers to indicate that the cost of pain mitigation is a factor benefit:cost cannot be the sole consideration when considering the care and welfare of farm animals.
